# Carbon-11 Radiotracing Reveals Physiological and Metabolic Responses of Maize Grown under Different Regimes of Boron Treatment

**DOI:** 10.3390/plants11030241

**Published:** 2022-01-18

**Authors:** Stacy L. Wilder, Stephanie Scott, Spenser Waller, Avery Powell, Mary Benoit, James M. Guthrie, Michael J. Schueller, Prameela Awale, Paula McSteen, Michaela S. Matthes, Richard A. Ferrieri

**Affiliations:** 1 Missouri Research Reactor Center, University of Missouri, Columbia, MO 65211, USA; wildersl@missouri.edu (S.L.W.); srstt9@mail.missouri.edu (S.S.); sgwxhv@mail.missouri.edu (S.W.); apgg4@mail.missouri.edu (A.P.); mvbf4w@mail.missouri.edu (M.B.); guthriejm@missouri.edu (J.M.G.); schuellerm@missouri.edu (M.J.S.); 2 School of Natural Resources, University of Missouri, Columbia, MO 65211, USA; 3 Division of Plant Sciences, University of Missouri, Columbia, MO 65211, USA; 4 Chemistry Department, University of Missouri, Columbia, MO 65211, USA; 5 Division of Biological Sciences, Bond Life Sciences Center, University of Missouri, Columbia, MO 65211, USA; pa96f@mail.missouri.edu (P.A.); mcsteenp@missouri.edu (P.M.); 6 Interdisciplinary Plant Group, University of Missouri, Columbia, MO 65211, USA; 7 Institute for Crop Science and Resource Conservation, Crop Functional Genomics, University of Bonn, Friedrich-Ebert-Allee 144, 53113 Bonn, Germany; mmatthes@uni-bonn.de

**Keywords:** carbon-11, boron effects, B73 *Zea mays*, inductively coupled plasma mass spectrometry

## Abstract

In agriculture, boron is known to play a critical role in healthy plant growth. To dissect the role of boron in maize metabolism, radioactive carbon-11 (t_½_ 20.4 min) was used to examine the physiological and metabolic responses of 3-week-old B73 maize plants to different levels of boron spanning 0 mM, 0.05 mM, and 0.5 mM boric acid (BA) treatments. Growth behavior, of both shoots and roots, was recorded and correlated to plant physiological responses. ^11^CO_2_ fixation, leaf export of [^11^C]-photosynthates, and their rate of transport increased systematically with increasing BA concentrations, while the fraction of [^11^C]-photosynthates delivered to the roots under 0 mM and 0.5 mM BA treatments was lower than under 0.05 mM BA treatment, likely due to changes in root growth. Additionally, solid-phase extraction coupled with gamma counting, radio-fluorescence thin layer chromatography, and radio-fluorescence high-performance liquid chromatography techniques applied to tissue extracts provided insight into the effects of BA treatment on ‘new’ carbon (as ^11^C) metabolism. Most notable was the strong influence reducing boron levels had on raising ^11^C partitioning into glutamine, aspartic acid, and asparagine. Altogether, the growth of maize under different regimes of boron affected ^11^CO_2_ fixation, its metabolism and allocation belowground, and altered root growth. Finally, inductively coupled plasma mass spectrometry provided insight into the effects of BA treatment on plant uptake of other essential nutrients. Here, levels of boron and zinc systematically increased in foliar tissues with increasing BA concentration. However, levels of magnesium, potassium, calcium, manganese, and iron remained unaffected by treatment. The rise in foliar zinc levels with increased BA concentration may contribute to improved ^11^CO_2_ fixation under these conditions.

## 1. Introduction

Crop yields are negatively impacted by various biotic and abiotic stresses and by nutrient deficiencies. Boron is a micronutrient essential for plant growth and development [1], affecting cell wall stability, plant metabolism, resource acquisition and allocation, and ultimately crop yield [2,3,4,5,6]. The range of soil boron concentrations for optimal plant growth is narrow and has been reported to be the narrowest among the micronutrients [7]. Both boron-deficient and boron-toxic soils can be found worldwide, overlapping with major plant growing areas and therefore negatively affecting many agricultural crops [2]. Both deficiency and toxicity of boron lead to impairments of plant growth and yield [8,9], necessitating studying how non-optimal boron conditions affect both plant development and metabolism.

Boron deficiency primarily leads to a cessation of growth in plant meristems (groups of undifferentiated stem cells) [10,11,12]. Belowground, shorter roots with fewer lateral roots are typically observed [12,13,14,15,16], while aboveground primarily reproductive tissues are affected by boron deficiency (as reviewed in [17]). Similar to boron deficiency, boron toxicity can lead to an inhibition of root growth [16,18,19,20,21,22] as well as causing leaf defects which manifest as necrotic regions, particularly at the leaf tips and the edges of the leaf blade [8,18]. This growth behavior is correlated with boron being passively transported via the transpiration stream and therefore accumulating at the end of the transpiration stream (i.e., the leaf tips and edges of the leaf blade) when in excess [23,24,25].

Boron can impact various metabolic and physiological processes (as reviewed in [3,17,26,27]) while the growth defects that develop in boron-deficient and -toxic conditions are causally linked with boron’s role in crosslinking two subunits of the pectin rhamnogalacturonan-II that contributes to the stabilization of the cell wall [28,29,30]. Since a plant’s requirement for boron is directly correlated with the pectin content in the cell wall, monocot species, like *Zea mays* (maize), have a lower boron requirement compared to dicotyledonous species [31]. Maize is one of the most important cereal crops worldwide, with the highest global annual yield [32]. Both boron deficiency and toxicity have detrimental effects on maize growth, development, and particularly yield [8,21,33,34].

Like other species, maize assimilates boron from the soil by the roots, through passive and active transport mechanisms [11,35,36,37]. Like many other plant species, boron deficiency in maize leads to shorter roots and a reduction of lateral root number [11,16,33,36]. However, the most prominent boron deficiency symptom in maize is a reduction of yield, due to the formation of small cobs with fewer kernels [34]. Reproductive tissues are more sensitive to boron deficiency compared to vegetative tissues, as seen by the phenotypes of maize transporter mutants [11,35,36,37]. Studies of boron toxicity on the physiological, metabolic, and developmental processes in maize are scarce [21,38,39,40]. Even so, these few studies have shown that exposure to high levels of boron in soil can inhibit root growth rates [40], resulting in reductions in root length [21] and root biomass [21,39,40] Additionally, boron toxicity can impair photosystem II efficiency [38], increase antioxidant activity [21,38], and generate chromosomal aberrations leading to genotoxic effects [40]. The reproductive defects observed under boron deficiency in maize can be rescued by boron supplementation [11,35,36,37,41] and by enhancing transpiration [41], suggesting that there is an impairment of physiological and metabolic processes related to transpiration and photosynthesis under boron deficiency.

Moreover, the negative effects of boron-deficient and boron-toxic conditions on plant photosynthesis (as reviewed in [5,42,43]), with widespread impacts on carbohydrate and amino acid metabolism [44,45,46,47,48], have been reported for other species. Even so, the mechanisms underlying these effects are unclear. Reductions in photosynthetic capacity in boron-deficient conditions were reported to be due to, for example, diffusion limits [49], diminished efficiency of electron transport [50], and reduced stomatal conductance which in turn reduces the photosynthetic rate as well as the rate of transpiration [51,52,53,54]. However, decreased levels of specific photosynthetic proteins have also been implicated in plants with boron deficiency effects [55]. Furthermore, reported negative effects on plant photosynthesis in boron-toxic conditions (as reviewed in [43]) have been attributed to both stomatal and non-stomatal limitations [22,56].

Altogether, it seems clear that the effects of boron deficiency and boron toxicity on carbohydrate and amino acid metabolisms can vary across species and studies. For example, both boron deficiency and toxicity can lead to the accumulation of non-structural carbohydrates according to some reports [47,48,52,57,58,59], as well as to their reduction in others [47,58,60,61]. Changes in amino acid metabolism also appears to depend on the type of boron stress applied, the tissue analyzed, and the plant species examined. For example, boron-deficient tobacco leaves exhibit enhanced glutamine levels compared to control leaves [62], while boron-deficient tobacco roots exhibit increased asparagine levels [62]. In boron-deficient citrus, asparagine accumulated, while amongst others less β-alanine and aspartate were detected [60]. Ascorbic acid and glutathione levels were decreased in boron-deficient squash root tips [63] and in shoot tips and young leaves of sunflower [42]. In boron-toxic *Arabidopsis* roots, the levels of multiple amino acids increased, including asparagine, glycine, glutamic acid, and proline [48].

It therefore appears that while boron deficiency and boron toxicity can often induce similar plant growth responses, the induced changes at a physiological and metabolic level can vary immensely across different plant species. Unfortunately, studies that span boron-deficient to boron-toxic growth conditions are scarce [47,55,56,64], as are studies analyzing the effects of boron on different tissue types [53,60,62].

To shed light on the previously unstudied effects of boron exposure on maize photosynthesis, carbohydrate, and amino acid metabolisms, we examined the developmental, physiological, and metabolic responses of 3-week-old B73 maize plants grown under different regimes spanning 0 mM boric acid (BA) treatment to 0.5 mM BA treatment, using ^11^C-radiotracing. This approach has proven to be powerful for examining plant responses to environmental stimuli [65] and in the present work provides unprecedented insight into the effects of boron on maize growth.

## 2. Results

**Figure 1:**
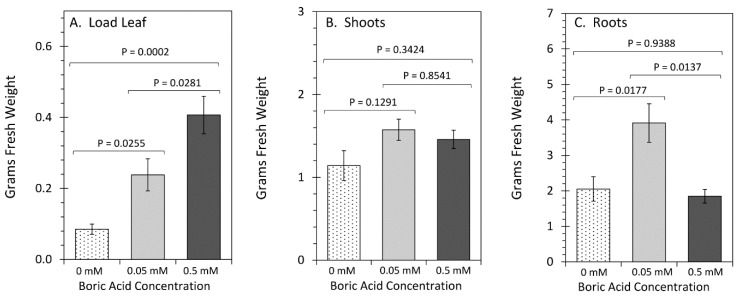
Fresh tissue masses for the load leaf ((**A**): leaf two at developmental stage V2 used to administer ^11^CO_2_), the shoots ((**B**): including all other leaves and stems), and roots (**C**) are presented here for plants grown under three regimes of boric acid (BA) treatment, including: 0 mM BA, 0.05 mM BA, and 0.5 mM BA. Levels of 0.05 mM BA corresponded to normal levels of boron found in Hoagland’s nutrient (see Table 1). Data bars represent average values ± SE on N = 8–10 replicates. Data was subjected to one-way analysis of variance with a post hoc Tukey’s HSD test to account for multiple comparisons between the different treatment groups (0 mM-to-0.05 mM BA; 0.5 mM-to-0.05 mM BA; and 0 mM-to-0.5 mM BA). Statistical significance was set at *p* < 0.05.

Plant Growth Responses to Boron: The effect of boron treatment on plant growth performance was examined across different tissue types. The results in Figure 1 reflect the changes observed in tissue masses (presented as grams fresh weight, gfw) for the load leaf (source leaf two, at V2 developmental stage, used to administer ^11^CO_2_), the shoots (including all other leaves and stems of the plant), and the roots from plants grown under three regimes of boric acid (BA) treatment: 0 mM BA, 0.05 mM BA, and 0.5 mM BA. We refer to 0.05 mM BA as normal boron levels, as this is the concentration of BA in Hoaglands solution used to grow the plants, while 0 mM BA refers to no added boron and 0.5 mM BA constitutes excess boron. Results showed a significant increase in load leaf mass from 0.09 ± 0.015 to 0.50 ± 0.081 gfw as boron levels were increased from 0 mM BA to 0.5 mM BA. Shoot masses were also significantly increased from 1.1 ± 0.1 to 1.6 ± 0.2 gfw with boron increasing from 0 mM BA to 0.05 mM BA but remained unchanged with further increase in BA. Likewise, root mass also significantly increased from 2.0 ± 0.2 to 4.0 ± 0.5 gfw as boron increased from 0 mM BA to 0.05 mM BA but then significantly decreased to 1.8 ± 0.2 gfw with 0.5 mM BA.

**Figure 2:**
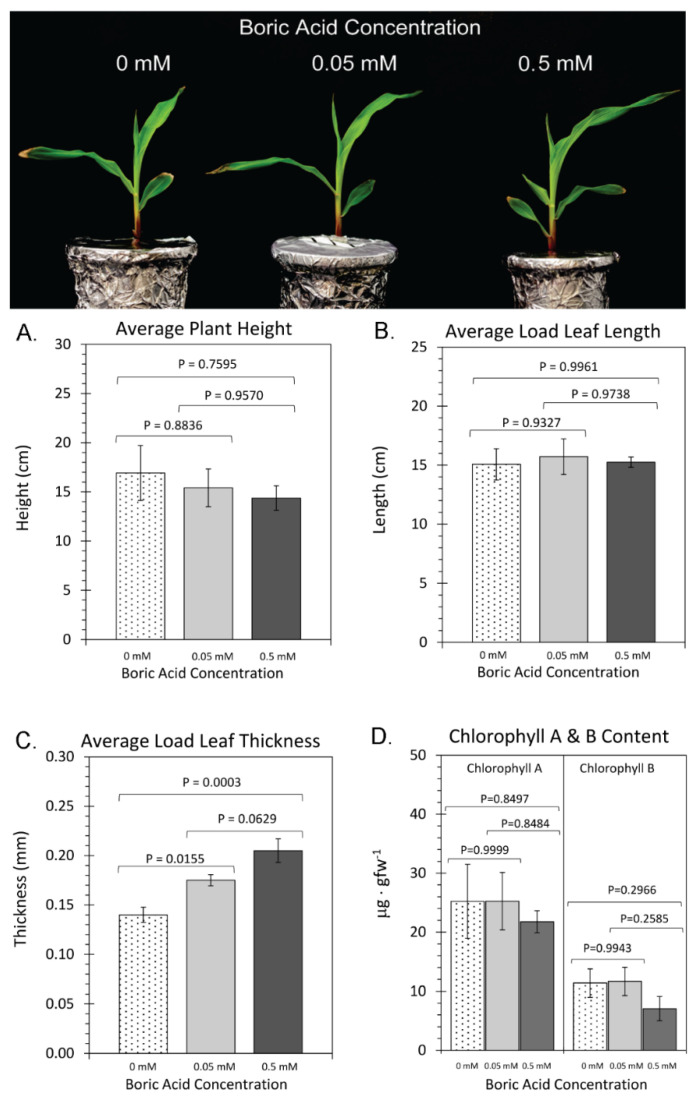
Shoot growth traits: upper panel depicts photos of plants that were grown under three regimes of boric acid (BA) treatment, including: 0 mM BA, 0.05 mM BA, and 0.5 mM BA. (**A**) depicts data on plant height measured in centimeters from the base of stem to the tallest leaf tip. (**B**) depicts data on the load leaf length (leaf two, at developmental stage V2, used to administer ^11^CO_2_) measured in centimeters. (**C**) depicts data on load leaf thickness measured in millimeters. (**D**) depicts data on load leaf chlorophyll A&B content measured in micrograms of chlorophyll per gram fresh weight of leaf tissue (μg⋅gfw^−1^). All data bars represent average values ± SE from N = 8–10 replicates. Data was subjected to one-way analysis of variance with a post hoc Tukey’s HSD test to account for multiple comparisons between the different treatment groups (0 mM-to-0.05 mM BA; 0.5 mM-to-0.05 mM BA; and 0 mM-to-0.5 mM BA). Statistical significance was set at *p* < 0.05.

To further dissect the causes of these changes in mass, additional measurements of shoot and root tissues were obtained. The levels of boron administered to the plant growth media had no effect on plant height (Figure 2A) nor on leaf length (Figure 2B). However, load leaf thickness (Figure 2C) significantly increased from 0.14 ± 0.01 to 0.205 ± 0.015 mm as boron levels were increased from 0 mM BA to 0.5 mM BA. Hand sections of leaves, followed by light and fluorescent microscopy, showed that there were no gross defects in Kranz anatomy or the number of cell layers that could explain the increase in thickness (Figure S4). Finally, leaf chlorophyll levels were unaffected by the level of boron introduced into the growth media. Chlorophyll A averaged around 25 μg⋅gfw^−1^ and was always significantly higher than chlorophyll B which averaged around 10 μg⋅gfw^−1^.

**Figure 3:**
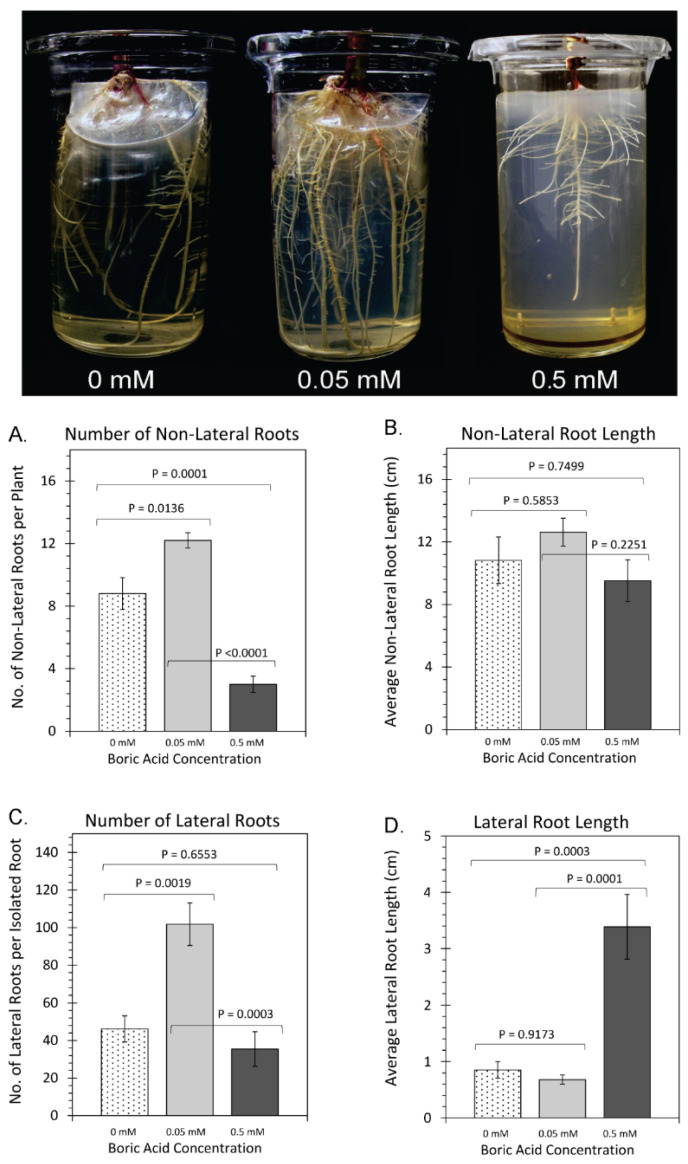
Root growth traits: upper panel depicts photos of plants that were grown under three regimes of boric acid (BA) treatment, including: 0 mM BA, 0.05 mM BA, and 0.5 mM BA. (**A**) depicts data on the average number of non-lateral roots (includes primary, seminal, and nodal roots) per plant. (**B**) depicts data on the average non-lateral root length measured in centimeters. (**C**) depicts data on the average number of lateral roots per root. (**D**) depicts data on the average lateral root length measured in centimeters. All data bars represent average values ± SE from N = 8–10 replicates. Data was subjected to one-way analysis of variance with a post hoc Tukey’s HSD test to account for multiple comparisons between the different treatment groups (0 mM-to-0.05 mM BA; 0.5 mM-to-0.05 mM BA; and 0 mM-to-0.5 mM BA). Statistical significance was set at *p* < 0.05.

In contrast to the effects of boron conditions on shoot growth, we observed drastic changes in root growth (Figure 3) across the different boron regimes. Both 0 mM BA and 0.5 mM BA treatments caused significant decreases in the number of roots (total of primary, seminal, and nodal roots) that grew (Figure 3A), decreasing from 12.0 ± 0.6 roots under normal boron conditions (0.05 mM BA) to 8.7 ± 1.0 roots and 3.0 ± 0.5 roots for growth under 0 mM BA and 0.5 mM BA, respectively. However, the average length of roots (Figure 3B) was not statistically significantly different with different BA treatments. The number of lateral roots exhibited similar phenotypic behavior to non-lateral roots. Both 0 mM BA and 0.5 mM BA treatments caused significant decreases in the number of lateral roots that could be detected (Figure 3C), decreasing from 104 ± 8 roots under normal boron conditions (0.05 mM BA) to 46 ± 5 lateral roots when grown under 0 mM BA and 36 ± 6 lateral roots when grown under 0.5 mM BA. In contrast, lateral root length (Figure 3D) remained unchanged with 0.8 ± 0.2 cm at 0 mM BA and 0.6 ± 0.1 cm at 0.05 mM BA but increased significantly to 3.4 ± 0.6 cm with 0.5 mM BA. Therefore, either increased or decreased boron led to a decrease in the number of lateral and non-lateral roots but had differing effects on the length of lateral and non-lateral roots.

**Figure 4:**
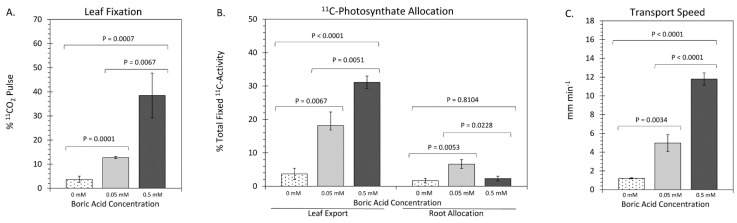
Plant physiological responses to growth under three regimes of boric acid (BA) treatment, including: 0 mM BA, 0.05 mM BA, and 0.5 mM BA. (**A**) depicts levels of ^11^CO_2_ fixation as the percent of ^11^CO_2_ shipped to the load leaf as a pulse normalized for differences in load leaf mass entrained within the leaf cuvette. The left side of (**B**) depicts levels of leaf export of ^11^C-photosynthates presented as percent total ^11^C activity fixed by the plant spanning 3 h after the radioactivity pulse was introduced to the load leaf. The right side depicts levels of ^11^C-photosynthates that arrived in the root tissue over the same 3 h period. (**C**) depicts the speed of transport of ^11^C-photosynthate in millimeters per minute (mm min^−1^), as labeled substrates moved across the two defined field-of-views shown in Figure S2. All data bars represent average values ± SE from N = 8–10 replicates. Data was subjected to one-way analysis of variance with a post hoc Tukey’s HSD test to account for multiple comparisons between the different treatment groups (0 mM-to-0.05 mM BA; 0.5 mM-to-0.05 mM BA; and 0 mM-to-0.5 mM BA). Statistical significance was set at *p* < 0.05.

Whole-Plant Physiological Responses to Boron: The impact of varying boron levels on leaf fixation of CO_2_, transformation to photosynthates and their export to roots was examined using ^11^C-radiotracing (Figure 4). Leaf fixation of ^11^CO_2_ (Figure 4A) increased significantly from 4.0 ± 1.5% with 0 mM BA to 38.0 ± 10.0% with 0.5 mM BA. Data was presented as percent of ^11^CO_2_ that was administered to the load leaf (leaf two at developmental stage V2) and was normalized to a 200 mg amount of leaf tissue exposed to tracer within the leaf cuvette. Leaf export of ^11^C-photosynthates as well as their allocation to roots was monitored over a 3 h period after initial exposure to tracer. Leaf export increased significantly from 3.2 ± 1.5% of fixed ^11^C at 0 mM BA to 31.5 ± 1.5% at 0.5 mM BA (Figure 4B). Allocation of ^11^C-photosynthates to roots did not behave in the same manner, but instead both 0 mM BA and 0.5 mM BA levels resulted in significant reductions of allocation to roots relative to normal boron levels of 0.05 mM BA, presumably due to the lower sink strength of reduced root mass (Figure 1 and Figure 3). Transport speed of ^11^C-photosynthates also changed significantly across the range of BA treatments, with speeds increasing from 1.21 ± 0.06 mm min^−1^ with 0 mM BA to 11.79 ± 0.66 mm min^−1^ with 0.5 mM BA.

**Figure 5:**
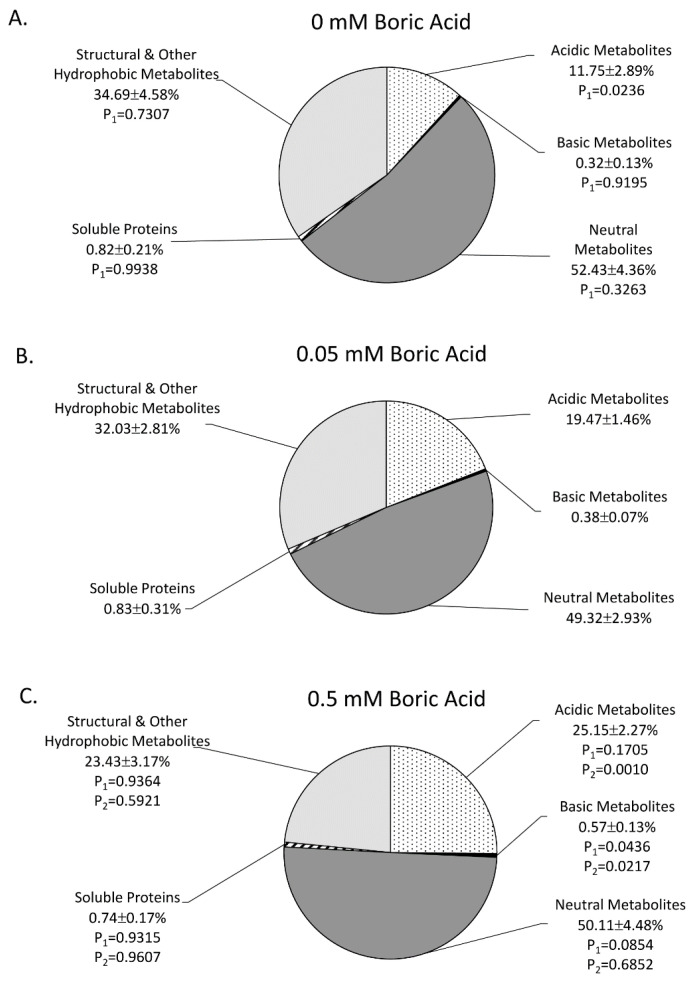
Metabolic partitioning of ‘new’ carbon across the load leaf’s different pools of substrates as a function of plant growth under three regimes of boric acid (BA) treatment, including: 0 mM BA (**A**), 0.05 mM BA (**B**), and 0.5 mM BA (**C**). Metabolite pools included: acidic, basic, and neutral metabolites as well as soluble proteins and a fraction representing hydrophobic and structural metabolites. Data represents percent yields based on percent ^11^C activity within the plant that were averaged across N = 8–10 replicates ± SE and then normalized to 100%. Data was subjected to one-way analysis of variance with a post hoc Tukey’s HSD test to account for multiple comparisons between the different treatment groups (0 mM-to-0.05 mM BA; 0.5 mM-to-0.05 mM BA; and 0 mM-to-0.5 mM BA). Statistical significance was set at *p* < 0.05, where P_1_ values denote comparisons of associated variable responses from 0 mM BA or 0.5 mM BA treatments to 0.05 mM BA, and P_2_ values denote cross comparisons between 0 mM BA and 0.5 mM BA treatments.

Effect of Boron on the Partitioning of ‘New’ Carbon into Leaf Metabolite Pools: ‘New’ carbon input as ^11^C into foliar metabolite pools often provides valuable insights as to where unfavorable growth conditions alter plant metabolism [16]. We therefore examined five distinct pools of ^11^C-metabolites, including the acidic, basic, neutral, and soluble protein fractions that were extractable in methanol:water, as well as structural/hydrophobic components that were not extractable in this solvent (Figure 5). The acidic metabolite fraction increased significantly in a BA dose-dependent manner from 11.75 ± 2.89% with 0 mM BA to 25.15 ± 2.27% with 0.5 mM BA. The rise in this pool was compensated by small reductions across all the other fractions.

**Figure 6:**
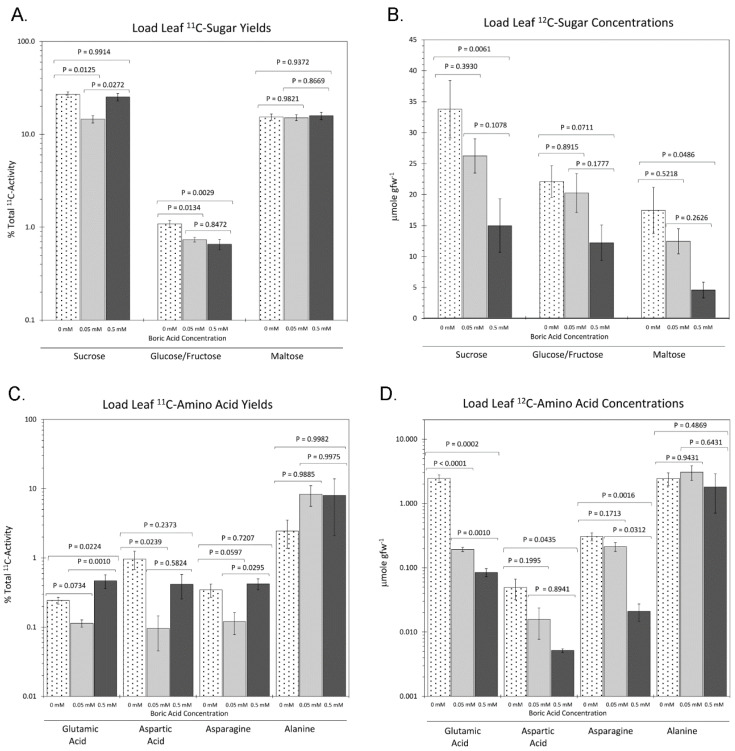
Individual ^11^C and ^12^C metabolite yields from the soluble sugar pool and amino acid pool as a function of plant growth under three regimes of boric acid (BA) treatment, including: 0 mM BA, 0.05 mM BA, and 0.5 mM BA. (**A**) depicts ^11^C-sugars yields measured in the load leaf 20 min after the administration of radioactivity. Data is presented as percent total ^11^C activity that the plant acquired from the pulse. (**B**) depicts the endogenous concentrations of the soluble sugars observed in the load leaf presented as micromoles of sugar per gram fresh weight of leaf tissue (μmol⋅gfw^−1^) used in the analysis. (**C**) depicts selected ^11^C-amino acid yields measured in the load leaf 20 min after administration of radioactivity. (**D**) depicts the endogenous concentrations of the same amino acids monitored in panel C within the load leaf presented as micromoles of sugar per gram fresh weight of leaf tissue (μmol⋅gfw^−1^) used in the analysis. All data represent average values ± SE from N = 8–10 replicates. Levels of significance are represented by *p* values calculated using one-way analysis of variance with a post hoc Tukey’s HSD test to account for multiple comparisons between the different treatment groups (0 mM-to-0.05 mM BA; 0.5 mM-to-0.05 mM BA; and 0 mM-to-0.5 mM BA). Statistical significance was set at *p* < 0.05.

Effect of Boron on ^11/12^C Isotope Distributions in Soluble Sugars and Key Amino Acids: The ^11/12^C isotope ratios of individual substrates within two distinct classes of plant metabolites were examined to shed further light on the effects of boron on plant metabolism. Figure 6A shows our examination of the input flux of ‘new’ carbon as ^11^C into sucrose, glucose/fructose, and maltose sugars. Results showed that growth under 0 mM BA and 0.5 mM BA caused significant increases in ^11^C-sucrose to 26.8 ± 1.81% and 25.18 ± 2.30%, respectively, from the 14.56 ± 1.32% level observed under normal boron growth (0.05 mM BA). Furthermore, 0 mM BA caused a significant increase in the ^11^C-labeled glucose/fructose fraction from 0.73 ± 0.04% under normal 0.05 mM BA conditions to 1.09 ± 0.09% under 0 mM BA, but growth under 0.5 mM BA did not alter this fraction relative to the normal boron control. Finally, ^11^C-maltose sugar was unaffected by the level of BA introduced into the growth media, where an average of approximately 15.5% was observed.

In addition, we assessed the effect of varying BA treatments on the ^12^C-endogenous pools of the same sugars (Figure 6B). The endogenous pools of sucrose, glucose/fructose, and maltose sugars all significantly declined across the increasing doses of applied BA to the growth media. Specifically, sucrose decreased from 33.8 ± 4.66 μmol gfw^−1^ at 0 mM BA to 14.99 ± 4.33 μmol gfw^−1^ at 0.5 mM BA, glucose/fructose decreased from 22.1 ± 2.56 μmol gfw^−1^ at 0 mM BA to 12.10 ± 2.85 μmol gfw^−1^ at 0.5 mM BA, though not statistically significantly, and maltose decreased from 17.44 ± 3.73 μmol gfw^−1^ at 0 mM BA to 4.59 ± 1.27 μmol gfw^−1^ at 0.5 mM BA.

We next examined the input flux of ‘new’ carbon as ^11^C into glutamic acid, aspartic acid, and asparagine, which reflects the aspartate regulation pathway, and alanine because it was the largest observed radiolabeled amino acid in the analysis (Figure 6C). While additional amino acids were detected, their yields were very small and therefore were omitted from the analysis. Growth under 0 mM BA and 0.5 mM BA caused significant increases in glutamic acid (0.24 ± 0.03% at 0 mM BA and 0.47 ± 0.11% at 0.5 mM BA relative to 0.11 ± 0.01% at 0.05 mM BA), in aspartic acid (0.97 ± 0.29% at 0 mM BA and 0.42 ± 0.16% at 0.5 mM BA relative to 0.09 ± 0.05% at 0.05 mM BA), and in asparagine (0.35 ± 0.07% at 0 mM BA and 0.42 ± 0.13% at 0.5 mM BA relative to 0.12 ± 0.04% at 0.05 mM BA) (Figure 6C). ^11^C-Alanine remained unaffected by the boron dose administered to the growth media at around 8% yield (Figure 6C). These results were complemented with assessing the effect of applied boron dose on the ^12^C-endogenous pools of the same amino acids (Figure 6D). The endogenous pools of glutamic acid, aspartic acid, and asparagine all significantly declined across the increasing doses of applied BA to the growth media. Specifically, glutamic acid decreased from 2.46 ± 0.33 μmol gfw^−1^ at 0 mM BA to 0.08 ± 0.01 μmol gfw^−1^ at 0.5 mM BA, aspartic acid decreased from 0.05 ± 0.02 μmol gfw^−1^ at 0 mM BA to 0.01 ± 0.0003 μmol gfw^−1^ at 0.5 mM BA, and asparagine decreased from 0.31 ± 0.04 μmol gfw^−1^ at 0 mM BA to 0.02 ± 0.006 μmol gfw^−1^ at 0.5 mM BA. ^12^C-Alanine levels remained unaffected at about 8 μmol gfw^−1^ by the applied boron doses.

**Figure 7:**
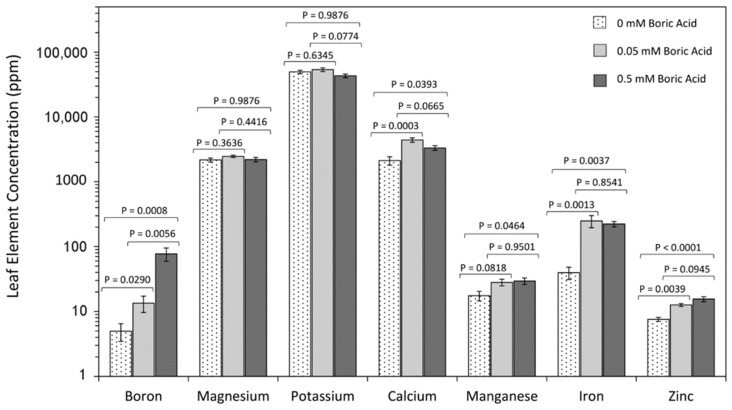
ICP-MS analysis of leaf tissues for elements, including boron, magnesium, potassium, calcium, manganese, iron, and zinc. Data bars reflect average values in ppm concentrations in dried tissue mass ± SE on N = 6 replicates. Levels of significance are represented by *p* values calculated using one-way analysis of variance with a post hoc Tukey’s HSD test to account for multiple comparisons between the different treatment groups (0 mM-to-0.05 mM BA; 0.5 mM-to-0.05 mM BA; and 0 mM-to-0.5 mM BA). Statistical significance was set at *p* < 0.05.

Effect of Boron on Foliar Elemental Distributions using Inductively Coupled Plasma-Mass Spectrometry (ICP-MS): To test the effect of boron treatment on the concentration of boron and other elements in leaf tissues, ICP-MS was used to quantify source leaf concentrations of essential cationic nutrients. In Figure 7, concentrations of boron, magnesium, potassium, calcium, manganese, iron, and zinc are shown quantified in parts per million (ppm) units. As expected, foliar boron levels rose significantly as the applied BA treatment increased, from 4.97 ± 1.51 ppm to 15.31 ± 3.46 ppm to 77.25 ± 17.86 ppm with 0 mM, 0.05 mM, and 0.5 mM BA respectively. These results correlate well with prior work showing that maize leaves typically contain 10–40 ppm boron [11,66] under standard growing conditions and that the boron sufficiency range for maize is 5–25 ppm [67].

As expected, other elements also showed changes across the range of BA treatments.

Zinc levels rose significantly from 7.57 ± 0.53 ppm at 0 mM BA to 15.53 ± 1.35 ppm at 0.5 mM BA. Potassium and magnesium levels appeared unaffected by boron levels across the full range of BA treatments examined. Calcium levels rose significantly from 2122.83 ± 312.35 ppm to 4416.67 ± 350.44 ppm with 0 mM and 0.05 mM BA treatments, respectively, and decreased to 3331.67 ± 273.87 ppm levels with 0.5 mM BA treatment, though the later change was not significant. Manganese and iron exhibited increasing levels from 17.50 ± 2.92 ppm to 28.07 ± 3.27 and 39.55 ± 8.52 to 248.17 ± 52.59 ppm, respectively, with 0 mM BA and 0.05 mM BA treatments. Therefore, boron treatments led to changes in some elements but not others.

## 3. Discussion

Boron has long been recognized as an essential micronutrient [1] affecting cell wall stability, plant metabolism, resource acquisition and allocation, and ultimately plant growth (as reviewed in [3,17,26,27]). While the primary role of boron in cell wall stability is accepted, what importance the additional effects of boron have is not resolved. This is partly due to the fact that the behavior of plants to varied levels of boron can differ significantly across the plant kingdom and even within the same species across the developmental stages of the plant, highlighting the importance of species-specific and developmentally well-defined studies. In the present work, we systematically examined plant growth characteristics, and measured the physiological and metabolic responses of maize plants grown under three regimes of boron, including 0 mM BA, 0.05 mM BA, and 0.5 mM BA.

Growth Responses to Boron: Although shoot traits were only subtly affected by varying BA treatments, we observed a decrease of load leaf thickness when plants were grown under 0 mM BA treatment and a systematic increase in thickness as the BA treatments were raised to 0.05 mM and 0.5 mM BA (Figure 2C). Our data implies that this trait was the sole reason for the observed rise in load leaf biomass with increasing BA concentrations (Figure 1). This result is in contrast to work in other species, where leaf thickness was reported to increase in boron-deficient conditions, including for example *Brassica napus* [49], due to the development of thicker cell walls in boron-deficient conditions [4]. Leaf sections indicated that there were no gross morphological defects; therefore, the reason for this increase in leaf thickness is predicted to be at the subcellular level.

Consistent with prior work in other species, shoot and root biomass was reduced in low boron conditions (Figure 1) [62,68]. In addition, we observed fewer roots (non-lateral and lateral) under 0 mM BA treatment as compared to normal boron levels at 0.05 mM BA treatment (Figure 3), which is consistent with previous boron deficiency studies in maize [16] and with the fact that root growth is more sensitive to boron influences than shoot growth (as reviewed in [69]). Furthermore, excessive levels of boron have also been shown to weaken roots, causing decreased root growth in *Arabidopsis*, wheat, barley, and maize plants [20,21,38,40,65,70]. We observed nearly the same root growth behavior under 0.05 mM BA treatment as with 0 mM BA treatment (Figure 3). Root numbers were lower under 0 mM and 0.5 mM BA treatments as compared with 0.05 mM BA treatment, in line with published results for, for example, applied boron toxicity in maize [16], boron-toxic barley, which showed reduced branching [71], and boron-toxic sunflower, where the number of adventitious roots decreased with increasing boron levels [72]. However, lateral root length significantly increased when grown under 0.5 mM BA, while it was unchanged when grown under 0 mM BA (Figure 3). To our knowledge there is no data available for lateral root lengths changes under varying boron conditions, yet root hair lengths was reported to increase in boron-deficient *Arabidopsis* (as reviewed in [73]). Making use of radioactively labelled phenylboronic acid, a boron-deficiency mimic, we previously showed that boron binding sites overlap with lateral root initiation sites [16], which could suggest that lateral roots need a high amount of boron for their development and/or their outgrowth in maize.

Sufficient concentrations of boron in maize have been reported to range from 5 to 25 ppm [16,66,67,74,75]. Since maize reportedly shows severe boron toxicity symptoms at tissue levels ranging from 50 ppm [38] to 100 ppm [76], our results of 77.25 ± 17.86 ppm (Figure 7) indicate that treatments using 0.5 mM BA were sufficient to produce high boron levels within the analyzed maize leaves that could be considered toxic in leaves. However, it nevertheless appears that the conditions applied in our experiment might not be toxic for lateral root development but rather beneficial. In the future, correlative analysis of boron measurements with growth kinetics on a tissue-specific scale spanning deficient to toxic boron growth conditions would resolve the reasons for boron effects on lateral root and non-lateral root initiation and growth. Furthermore, as the number of primary and seminal roots are determined during embryogenesis, the effects on the number of roots seen in our conditions, presumably primarily affected nodal roots, which are the major root type during most maize growth and development stages.

An alternative explanation of the increasing lateral root length in excess boron conditions, lies in the fact that foliar zinc levels systematically increased with increasing BA concentrations (Figure 7). Consistent with the results presented here, elevated zinc levels have been reported in boron-deficient mulberry leaves [77]. The biosynthesis of the phytohormone auxin in plants and zinc levels are strongly correlated [78,79,80], and auxin plays a strong role in regulating lateral root growth [32,81]. With tryptophan being the principal intermediate in auxin biosynthesis, withholding zinc was shown to lower plant tryptophan levels [80] and auxin levels [78], while exogenous treatment with zinc increased tryptophan levels [82]. Hence, the lateral root growth response seen under excess boron could be attributed to the promotion of zinc uptake and an increase in auxin levels.

While zinc was the only micronutrient that systematically increased with increasing boric acid treatment, we did observe specific changes of the other analyzed nutrients across the range of boric acid treatments (Figure 7). Calcium levels were decreased under 0 mM BA and 0.5 mM BA treatments. The results obtained for 0 mM BA maize leaves were in line with prior results reported for boron-deficient tobacco leaves [83] and with results from boron-toxic maize leaves [38]. It is interesting to note that calcium has been reported to be associated with sensing boron deficiency in plants and that calcium levels increased in boron-deficient tobacco BY-2 cells [84], *Arabidopsis* roots [85,86], and *Malus domestica* pollen tips [87]. These contrasting results could imply that calcium is not involved in a process sensing boron in leaves in maize. Contrary to this, manganese and iron levels decreased in the 0 mM BA treated leaves compared to control (Figure 7). The results for manganese and iron are in contrast to results for Mulberry, where iron and manganese concentrations appeared elevated in boron-deficient leaves [77]. Previously, we observed decreased manganese concentrations and unaltered iron concentrations in boron-deficient maize roots [16]. While these contrasting results highlight species- and tissue-specific differences in boron stress responses, they also suggest a connection between boron and manganese levels in maize that should be explored in future studies [3].

Plant Physiological Responses to Boron: It is well-documented that boron will affect the photosynthetic capacity of plants, impacting the transport of photosynthetic products to sink tissues [26,51,56,88,89]. We observed a systematic rise in ^11^CO_2_ fixation with increasing boron as well as a systematic rise in leaf export of ^11^C-photosynthates, all transporting at faster rates through the phloem (Figure 4), indicating a negative effect of lower boron levels on photosynthesis in maize and a positive effect of excess boron on these photosynthesis traits. While the negative effects of boron deficiency on photosynthesis have been seen in previous findings in various species (as reviewed in [42,69]), positive effects on photosynthesis by excess boron conditions have to the best of our knowledge not been reported previously (as reviewed in [43]). This might be due to the following: (i) only few studies have looked at the effects of varying boron concentration on maize photosynthetic traits [21,38]; (ii) the use of radioactive carbon-11 in combination with powerful radioanalytical tools to assess boron stress responses regarding maize photosynthesis are unprecedented; and (iii) maize uses C4 photosynthesis, which might be affected differently by boron stresses compared to published work on C3 species. The three traits (^11^CO_2_ fixation, leaf export of ^11^C-photosynthates, and their transport rate) are mutually entwined—as the plant takes up more carbon via leaf photosynthesis, the supply of mobile photosynthates increases, driving dynamic transport to a higher level.

On the other hand, allocation of ^11^C-photosynthates to root sink tissues did not follow the same pattern of behavior, likely due to the fact that root growth was severely impacted by the amount of boron in the growth media (Figure 3). The question remains as to what is driving the increased rate of photosynthesis as boron levels are increased. We noted earlier that growth under excess boron caused thicker leaves to develop, although the data in Figure 4A was normalized to a fixed leaf biomass entrained within the leaf cuvette. Therefore, we cannot explain the results based on biomass. In addition, there were no significant changes in leaf anatomy (Figure S4). Furthermore, we noted that load leaf chlorophyll content (Figure 2D) was unaltered by the amount of boron in the growth media, which implies that the increase in photosynthetic capacity with increasing BA supply in maize cannot be explained based on changes in foliar chlorophyll. Previous reports about chlorophyll content in boron stress conditions are inconclusive. For example, in boron-deficient conditions, chlorophyll content in *Arabidopsis* leaves was significantly increased [55], while in boron excess conditions it was significantly decreased [55]. Contrary to this, boron-deficient growth caused the chlorophyll content in olive leaves to be significantly lower than that observed under normal growth conditions [57], while excess boron conditions in maize did not affect the chlorophyll content of leaves relative to normal growth conditions [38]. Altogether, the contrasting results of prior published studies suggests that more work is needed to elucidate the effects of boron supply on chlorophyll regulation.

As noted earlier, foliar zinc levels increased systematically with increases in boron (Figure 7). Past studies in cauliflower have shown that a reduction in photosynthesis induced by zinc deficiency was associated with a decrease in stomatal conductance and intercellular CO_2_ concentration [90]. Furthermore, a decrease of carbonic anhydrase activity due to foliar zinc levels may have also contributed to the reduced net photosynthetic rate [91,92,93,94]. It has been shown that a higher net photosynthetic rate could be obtained in a zinc deficiency-resistant wheat cultivar than in a zinc-sensitive cultivar that was related to higher carbonic anhydrase activity [95]. However, other studies [96] suggest that zinc affects stomatal conductance. Hence, the inter-relationship between zinc, boron, and leaf photosynthesis is not a simple one and not yet fully understood.

Plant Metabolic Responses to Boron: Boron’s primary role in plant metabolism is the stabilization of molecules with *cis*-diol groups, as shown for Rhamnogalacturonan-II in the cell wall [28,29,30]. Additionally, boron can form complexes with glycoproteins and glycolipids within the plasma membrane [97,98]. In doing so, boron is implicated in the regulation of many enzyme-driven reactions as well as in the intercellular trafficking of ions, metabolites, and plant hormones [3,26]. Since boron impacts the photosynthetic capacity of the plant, changes in intercellular CO_2_ concentrations can result in oxidative damage [51]. To maintain a net carbon balance under boron extremes, plants will up-regulate leaf respiration, organic acid metabolism, and amino acid biosynthesis as compensatory measures [52,53]. Indeed, the present studies showed a significant reduction in ‘new’ carbon fluxes into the organic acid pool, which increased systematically as the level of applied BA increased (Figure 5). This response was partially compensated by the systematic change seen in the hydrophobic and structural metabolite fraction. It has been noted that under boron deficiency, levels of phenolic metabolites can become elevated in leaves [99]. Though we did not attempt to analyze specific metabolites within this fraction, we expect that such compounds can contribute heavily to the hydrophobic constituents that make up lignin.

Our observation of heightened ‘new’ carbon (as ^11^C) residing in the soluble sugar pools (Figure 6) under low boron conditions correlates well with the expectation that, overall, there is a loss in capacity for the plant to build cell walls causing a backlog of sugar resources. We detected an increase of ^11^C-sucrose in both 0 mM BA and 0.5 mM BA maize leaves, while an accumulation of ^11^C-glucose/fructose was only seen in 0 mM BA maize leaves (Figure 6A). The systematic increase in leaf thickness with increasing BA treatment may explain why we additionally observed an upturn in ^11^C partitioning into sucrose with excess boron. As the demand for sugars supplying cell wall construction increased, the metabolic machinery regulating the partitioning of ‘new’ carbon into these pools likely followed. Most notable is the fact that the endogenous pools of these same soluble sugars systematically decreased with increasing BA concentrations (Figure 6B), showing differential responses to endogenous sugar concentrations with different BA treatments in maize leaves. We detected an accumulation of sucrose, glucose/fructose, and maltose in 0 mM BA treatment maize leaves, similar to what has been reported in cotton [58], tobacco [59], citrus [100], and birch [47], amongst others. In contrast, sucrose, glucose/fructose, and maltose were significantly reduced in excess boron maize leaves (Figure 6B). Similar observations were made in birch seedlings [47], while in *Arabidopsis* boron-toxic shoots an increase in several sugars, including fructose and glucose, was detected [48]. Our data therefore implied a backlog of sugar resources in low boron conditions and suggested that as the plant’s capacity to build new cells walls increases, its consumption of sugars rises.

Finally, the present work showed clear patterns of response to boron regarding the status of certain amino acids. Firstly, both the input of ‘new’ carbon (as ^11^C) into alanine as well as its endogenous concentration were extremely elevated throughout the studies and appeared unaffected by the level of applied BA. We note that past studies have associated plant hypoxia-induced stresses with alanine accumulation [101] and therefore believe that the nature in which our maize plants were grown may have contributed to an overarching hypoxia-induced stress causing this elevation. Even so, this stress was presumed constant throughout all the BA treatments. We detected significant elevations for the input ‘new’ carbon fluxes into glutamic acid, aspartic acid, and asparagine with both low and high boron treatments relative to the control treatment (Figure 6C). Given how these three amino acids are intertwined in essential plant metabolism (for a review, see [102]), it seems plausible that their metabolic regulation would be similar in response to boron and especially to boron deficiency [103]. Like the sugars, similar metabolic behaviors exhibited here involving the upregulation of ‘new’ carbon fluxes under boron deficiency and excess boron conditions are likely due to different mechanisms of action and different plant requirements of these amino acids. We know that many amino acids can regulate multiple processes related to gene expression, not only on a global scale but also by inducing preferential translation of mRNA encoding particular proteins [104]. Enhanced asparagine levels have been connected to effects of boron deficiency on nitrate assimilation [46,62]. Enhanced asparagine levels were observed in boron-deficient tobacco roots [46,62], which were proposed to be due to a role of boron levels in the assimilation of ammonium by asparagine synthetase. In fact, under low boron conditions we saw elevated levels in the endogenous concentrations of glutamic acid, aspartic acid, and, most importantly, asparagine (Figure 6D). These results contrast with studies in tobacco and citrus leaves, where asparagine levels were decreased in boron-deficient conditions [46,83,100]. They could, however, imply a similar role of boron levels in nitrate assimilation in maize leaves, as proposed for tobacco roots. As the plant boron levels increased, the endogenous levels of glutamic acid, aspartic acid, and asparagine systematically decreased (Figure 6D), either because a potential role of boron in nitrate assimilation is specific for boron deficiency or their utilization in other plant metabolic processes was promoted.

## 4. Materials and Methods

Plant Growth: Kernels of the B73 line of maize (USDA Agricultural Resource Services-Germplasm Resource Network, Ames, IA, USA) were germinated in darkness for 48 h in Petri dishes containing sterile paper towels wetted with 2% slurried sodium bicarbonate in water. The Petri dishes were kept in the dark at 30 °C for 48 h after which they were relocated to a commercial growth chamber (Model AR-66L2, Percival Scientific, Perry, IA, USA) giving 100 μmol m^−2^ s^−1^ of light intensity during a 12 h photoperiod. When the primary root of the germinating seedling reached 1–2 cm in length, the seedling was transplanted into a 600 mL Pyrex glass growth cell filled with Hoagland’s fortified GelRite™ gellan gum gel.

Gels were prepared in 3 L batches of deionized water (18 MΩ) mixed with micronutrient salts (Table 1) to make up Hoagland’s reagent plus 1.66 g MES hydrate at three levels of boric acid.

Once mixed, the pH levels of the solutions were adjusted to 6.0 using 30% sodium hydroxide. After pH adjustment, 8.4 g GelRite™ (Sigma-Aldrich, St. Louis, MO, USA) was added and the solutions were autoclaved at 120 °C followed by high-speed vortex mixing for 2 h to enable aeration of the viscous solution before it had a chance to gel. Prior to solidification, the 600 mL Pyrex glass cells were filled and covered to prevent desiccation.

Once transplanted to gels, seedlings were placed in a commercial growth chamber (Model PGC-15, Percival Scientific, Perry, IA, USA) with growth conditions of 12 h photoperiods, 500 μmol m^−2^ s^−1^ light intensity, and temperatures of 25 °C/20 °C (light/dark) with humidity at 70–80% for three weeks.

Plant Growth Behavior: Aboveground tissues were examined for growth characteristics which included measurement of plant height from the base of the stem to the tip of the longest leaf. Precision calipers (Fowler High Precision, Newton, MA, USA) were used to determine the thickness of load leaf tissue. Leaf two was used to administer ^11^CO_2_ to the plant at developmental stage V2 [105].

Roots subjected to the described growth conditions were harvested from the gel matrix, photographed using a DSLR camera, and weighed for fresh mass of tissue. Additionally, isolated roots were removed from the total biomass, suspended in a tray of water and re-photographed (Figure S1). Suspending the root in water allowed the lateral roots to separate. Root photographs were processed using AmScope v4.11.18421 software (AmScope, Inc., Irvine, CA, USA) to determine the average length of roots, including primary, seminal and any nodal roots [32], as well as the number and average length of lateral roots.

Chlorophyll Measurements: Previously frozen leaf 2 samples from V2 stage plants were placed in pre-weighed, pre-chilled Eppendorf™ tubes, flash frozen in liquid nitrogen, ground to a fine powder, and weighed. A volume of acetone equivalent to four times the milligram mass of the frozen ground tissue was added to the centrifuge tube. The content was vortexed (VWR analog vortex mixer; Sigma-Aldrich Corp., St. Louis, MO, USA) then sonicated (Branson Bransonic 32; Sigma-Aldrich Corp., St. Louis, MO, USA) at 0 °C for 2 min. Additional vortexing was performed during this period to ensure complete mixing. The Eppendorf™ tubes were then centrifuged for 2 min at 15,000 rpm to separate the insoluble and soluble portions. The insoluble portion contained mostly cell wall polymers and starch while the soluble portion contained small soluble compounds, such as sugars. The liquid extract was spotted onto a silica TLC plate in a 5 mm band using 2 μL of sample. The silica plate was developed in a 6.0:1.6:1.0:0.4 ratio by volume of petroleum ether:hexane:acetone:methanol solution. Once the plate was fully developed, a razor was used to scrape off bands containing chlorophyll A and chlorophyll B. The shavings were placed in two separate Eppendorf™ tubes and acetone (1 mL) was added to each tube. The samples were then vortexed and centrifuged for 4 min at 15,000 rpm. The absorbance of the resulting supernatant of each sample was analyzed via ultraviolet–visible spectroscopy (UV–Vis). Chlorophyll A was recorded at a wavelength of 663 nm, and Chlorophyll B was measured at a wavelength of 645 nm. Beer’s Law (A = ε bc) was then utilized to determine the sample concentration of chlorophyll.

*Leaf anatomy:* Leaf 3 samples of three-week-old plants grown in different levels of BA were removed and stored in ethanol. Hand sections were cut with a razor blade from the middle (widest) part of the leaf, excluding the midvein. Sections were floated in water, covered with a coverslip, and viewed using bright field and UV fluorescence microscopy with an Olympus BX61 microscope (Olympus Corp., Tokyo, Japan) equipped with an XC10 CCD digital camera (Olympus America, Inc., New York, NY, USA).

Production and Administration of Radioactive ^11^CO_2_: ^11^CO_2_ (t_½_ = 20.4 min) was produced on the GE 800 Series PETtrace Cyclotron located at the Missouri Research Reactor Center using high-pressure research-grade N_2_ gas target irradiated with a 16.4 MeV proton beam to generate ^11^C via the ^14^N(p,α)^11^C nuclear transformation [106,107]. The ^11^CO_2_ was trapped on the molecular sieve, desorbed, and quickly released into an air stream at 200 mL min^−1^ as a discrete pulse for labeling a leaf affixed within a 5 × 10 cm lighted (560 μmol m^−2^ s^−1^) leaf cell to ensure a steady level of fixation. The load leaf (source leaf two, at developmental stage V2) affixed within the cell was pulse-fed ^11^CO_2_ for 1 min in a stream of air at 200 mL min^−1^. A PIN diode radiation detector (Carroll Ramsey Associates, Berkeley, CA USA) attached to the bottom of the leaf cell enabled continuous measurement of radioactivity levels within the cell during the initial pulse and in the minutes directly following to give information on ^11^CO_2_ fixation and leaf export of [^11^C]-photosynthates [108].

Whole-Plant [^11^C]-Physiology Measurements: After ^11^CO_2_ pulsing, plants were incubated for 3 h before separating the load leaf from shoots and roots. During that time, levels of radioactivity were monitored using two radiation detectors (Eckler & Ziegler, Inc., Berlin, Germany 1-inch Na-PMT, photomultiplier tube gamma radiation detector) affixed to the plant 8 cm above the base of the stem and below the base of the stem (Figure S2) which provided dynamic feedback on ^11^C-photosynthate transport. Data were acquired at a 1 Hz sampling rate using 0–1 V analog input into an acquisition box (SRI, Inc., Torrance, CA, USA). Measurement of ^11^C radioactivity was performed using gamma counting and data was decay-corrected to end of bombardment. The individual components were summed together for total plant ^11^C radioactivity and normalized based on fresh tissue weight. Individual components were used to calculate leaf export and root allocation fractions.

[^11^C]-Metabolite Analyses: After ^11^CO_2_ pulsing, the load leaf was removed 20 min later and subjected to metabolite analyses following published procedures [109]. Tissue was flash frozen in liquid nitrogen, ground to a fine powder, and extracted in methanol:water (60:40 *v*/*v*) via sonication (Branson, Bransonic 32; Sigma-Aldrich Corp., St. Louis, MO, USA) for 2 min at 100% amplitude in Eppendorf™ tubes. After centrifugation at 15,000 rpm for 2 min the insoluble and soluble portions were separated. The soluble extract supernatant was placed in a separate Eppendorf™. A 20 µL aliquot of the soluble extract and the entire insoluble pellet were counted for ^11^C radioactivity using a NaI gamma counter. The insoluble portion contained mostly cell wall polymers, starch, and other hydrophobic metabolites. The soluble portion contained small water-soluble compounds, including sugars, amino acids, and non-nitrogen-containing organic acids. All data was decay-corrected back to the end of bombardment or end of cyclotron beam.

Total soluble [^11^C]-sugars were measured by radio thin layer chromatography (TLC) using glass-backed NH_2_-silica HPTLC plates (200 µm, w/UV254) purchased from Sorbent Technologies (Atlanta, GA, USA) according to published procedures [110]. A mobile phase consisting of 65:20:15 acetonitrile:methanol:deionized water (*v*/*v*) was used. After development, TLC plates were imaged using autoradiography on a Typhoon 9000 imager (TyphoonTM FLA 9000, GE Healthcare, Piscataway, NJ, USA) and radioactivity was quantified using ImageQuant TL 7.0 software. Total [^11^C]-sugar content was related to the ^11^C radioactivity quantified along the sample lane of the TLC plate and then corrected to percent total fixed ^11^CO_2_ using gamma count data from the insoluble and soluble fractions.

[^11^C]-amino acids were analyzed following published procedures [109] using pre-column OPA derivatization of 100 µL of the methanol:water extract (1:1 ratio) and quantified by gradient radio HPLC (Sonntek, Inc., Upper Saddle River, NJ, USA). The method used a Phenomenex Gemini 5 µm C18 (150 mm × 4.6 mm inner diameter) column heated to 30 °C and a mobile phase system comprising Solvent A (95:5 *v*/*v* with 0.5 M sodium acetate:methanol) and Solvent B (70:30 methanol:18 MΩ water) starting at 75:25 and switching to 25:75 within 30 min at a flow rate of 0.7 mL min^−1^. On-line fluorescence detection (340 nm excitation, 450 nm emission; Hitachi LaChrom Elite L-2485; Sonntek, Inc., Upper Saddle River, NJ, USA) was used for correlating retention times of standards with those of [^11^C]-amino acids in biological samples. A NaI gamma radiation detector (Ortec, Inc., Oak Ridge, TN, USA) enabled direct measurement of radiolabeled metabolites. Data was acquired using PeakSimple™ chromatography software (SRI, Inc., Torrance, CA, USA) and radioactivity assigned to peaks was corrected for radioactive decay, summed for a total [^11^C]-amino acid value, and related back to the amount of ^11^C radioactivity fixed by the plant at the start of the study (Figure S3).

For [^11^C]-organic acid analysis, 500 µL of leaf extract was rendered slightly basic (pH 8.5) using 1 N NaOH, and the total volume was processed through an Accell QMA Plus Light Sep-Pak™ (Waters Corporation, Milford, MA, USA) followed by rinsing of the contents with 10 mL of DI water. Cartridges were then counted for ^11^C radioactivity with a NaI gamma counter.

For [^11^C]-basic metabolite analysis, 200 mL of tissue extract was processed through a strong cation exchange cartridge (Strata-XL-C, Phenomenex, Inc., Torrance, CA, USA) followed by rinsing of the cartridge using 10 mL of DI water. The cartridge was subjected to gamma counting for quantification of this metabolite pool.

Inductively Coupled Plasma-Mass Spectrometry (ICP-MS): For ICP-MS analyses of nutrients, leaf 2 samples were harvested at the V2 developmental stage from plants, and 1 cm of leaf tips and lower sheath regions were removed. Tissues were air dried at 70 °C for 1 week and ground to a powder using a mortar and pestle. Ground tissue was weighed and digested in 3.0 mL of concentrated nitric acid at 190 °C using a Milestone Ethos Plus (Milestone SRL, Sorisole, Italy) microwave digestion system, then diluted to 50 mL with ultrapure water followed by gravimetric dilution by a factor of 10 with 0.45 N nitric acid. Samples were analyzed with a Perkin-Elmer NexION ICP-MS in Kinetic Energy Discrimination mode. Reference materials included NIST SRM 1570 spinach leaves and NIST SRM 1573 tomato leaves prepared in the same way. Internal standards at known concentrations were prepared from stock solutions (High Purity Standards, Charleston, SC, USA) and used to calibrate instrument response.

Statistical Analysis: Data was subjected to the Shapiro–Wilk Normality Test to identify outliers, so all data groups reflected normal distributions. Data was then subjected to one-way analysis of variance (ANOVA) in R using the multcompview package [111]. Tukey’s HSD test was used for post hoc correction for multiple comparisons between the different treatment groups (0 mM-to-0.05 mM BA; 0.5 mM-to-0.05 mM BA; and 0 mM-to-0.5 mM BA) at a significance level of *p* < 0.05.

## 5. Conclusions

Using a combination of radiotracer tools and ICP-MS, we were able to gain new insight into the physiological and metabolic responses of maize plants subjected to different levels of BA treatment during growth spanning low to high boron levels. ^11^C-radiotracing allowed us to quantify changes in the plants’ physiological status by examining changes in ^11^CO_2_ fixation, leaf export of ^11^C-photosynthates, their rate of transport, and the amount of photosynthates that were allocated to roots. Furthermore, these biological functions were correlated with the growth performance of plants, both aboveground and belowground, as well as correlated with plant uptake of essential nutrients. Finally, ^11^C-radiotracing provided a way to examine changes in metabolic regulation by measuring ^11^C-partitioning across all the plant metabolite pools and by measuring certain individual metabolites within these pools. Our study therefore provides unprecedented insight into the effects of boron on maize physiological and metabolic processes. By examining the flux of ‘new’ carbon (as ^11^C) into metabolites, one can often gain greater insight into boron’s influence on plant metabolism and its regulation than by simply profiling the endogenous metabolite concentrations. Most notable here were the strong influence that reducing boron levels had on raising ^11^C partitioning into glutamine, aspartic acid, and asparagine, which may be part of a plant stress response to boron deficiency.

Finally, inductively coupled plasma mass spectrometry provided another layer of insight into the effects of boron on plant uptake of essential micronutrients. Here, levels of boron and zinc were seen to systematically increase in foliar tissues with increasing BA concentration, while levels of magnesium, potassium, calcium, manganese, and iron remained unaffected by treatment. This rise in foliar zinc levels with increased BA concentrations may contribute to improved ^11^CO_2_ fixation under these conditions. Altogether, boron appears to have a selective effect on the uptake of only certain plant micronutrients. Further studies will be needed to elucidate the mechanisms for this behavior.

## Figures and Tables

**Table 1:** Nutrient Composition.

Micronutrients		Concentration
KNO_3_		6.0 mM
CaCl_2_		2.0 mM
KH_2_PO_4_		2.0 mM
MgSO_4_		2.0 mM
FeEDTA Solution 1 M KOH 1.04% EDTA∙2Na 0.78% FeSO_4_∙7H_2_O	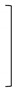	0.078%
MnCl_2_∙4H_2_O		9.1 µM
ZnSO_4_∙7H_2_O		0.76 µM
CuSO_4_∙5H_2_O		0.32 µM
NaMoO_4_∙2H_2_O		0.50 µM
H_3_BO_3_ (3 levels)		0 mM, 0.05 mM, 0.5 mM

## Data Availability

Data available upon request from the authors.

## Supplementary Materials

The following supporting information can be downloaded at: https://www.mdpi.com/article/10.3390/plants11030241/s1, Figure S1: Root growth traits; Figure S2: Experimental setup for measuring transport of ^11^C-photosynthates across plant tissues; Figure S3: Radio HPLC traces depicting ^11^C-amino acids; Figure S4: Brightfield and fluorescence images of leaf cross sections.
