# Supplementary material for: Carbon-11 Radiotracing Reveals Physiological and Metabolic Responses of Maize Grown under Different Regimes of Boron Treatment
Source: Plants (Basel). 2022 Jan 18;11(3):241. doi: 10.3390/plants11030241 (PMC8839955; doi:10.3390/plants11030241)
Supplement: Supplementary file 1 [file plants-11-00241-s001.zip › plants-1507314-SI.pdf]

Supplemental Material

Carbon-11 Radiotracing Reveals Physiological and Metabolic Responses of Maize Grown under Different Regimes of Boron Treatment

**S. L. Wilder [1,†], S. Scott [1,†], S. Waller [1,2], A. Powell [1,2], M. Benoit [1,3], J. M. Guthrie [1], M. J. Schueller [1,4], P. Awale [5,6],
P. McSteen [5,6], M. S. Matthes [7] and R. A. Ferrieri [1,3,4,6,*]**

1 Missouri Research Reactor Center, University of Missouri, Columbia, MO 65211, USA; wildersl@missouri.edu (S.L.W.); srstt9@mail.missouri.edu (S.S.); sgwxhv@mail.missouri.edu (S.W.); apgg4@mail.missouri.edu (A.P.); mvbf4w@mail.missouri.edu (M.B.); guthriejm@missouri.edu (J.M.G.); schuellerm@missouri.edu (M.J.S.)
2 School of Natural Resources, University of Missouri, Columbia, MO 65211, USA
3 Division of Plant Sciences, University of Missouri, Columbia, MO 65211, USA
4 Chemistry Department, University of Missouri, Columbia, MO 65211, USA
5 Division of Biological Sciences, Bond Life Sciences Center, University of Missouri, Columbia, MO 65211, USA; pa96f@mail.missouri.edu (P.A.); mcsteenp@missouri.edu (P.M.)
6 Interdisciplinary Plant Group, University of Missouri, Columbia, MO 65211, USA
7 Institute for Crop Science and Resource Conservation, Crop Functional Genomics, University of Bonn, Friedrich-Ebert-Allee 144, 53113 Bonn, Germany; mmatthes@uni-bonn.de (M.S.M)
* Correspondence: ferrierir@missouri.edu; Tel.: +1-573-882-5211
† These authors have contributed equally to this work and share first authorship.

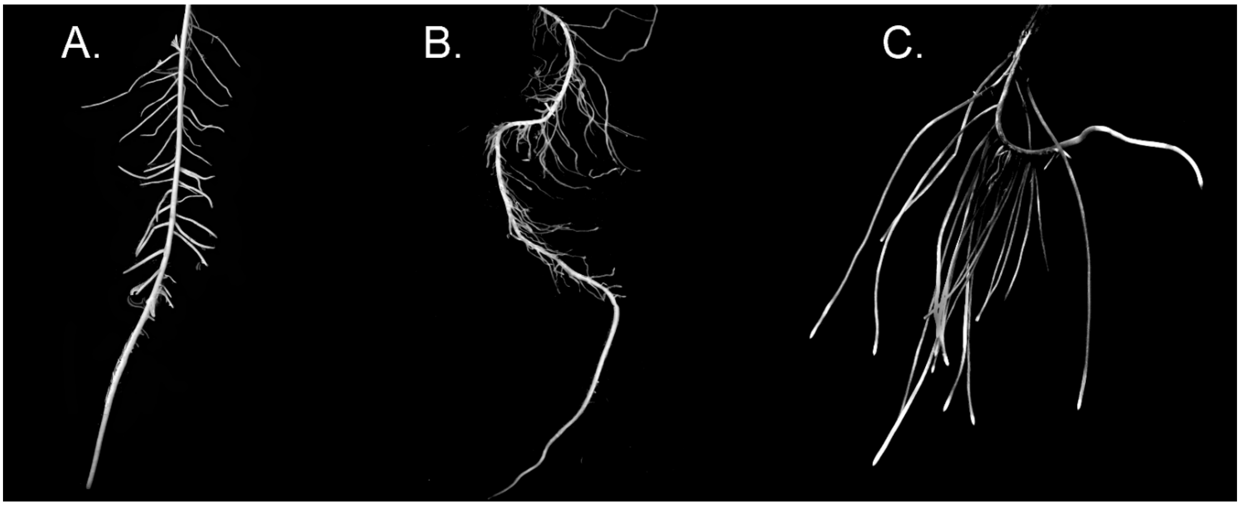

Figure S1. Isolated roots from plants grown under 0 mM boric acid (Panel A), 0,05 mM boric acid (Panel B) and 0.5 mM boric acid (Panel C) were floated in a tray of water, digitally photographed, and subjected to root analyses using AmScope software.

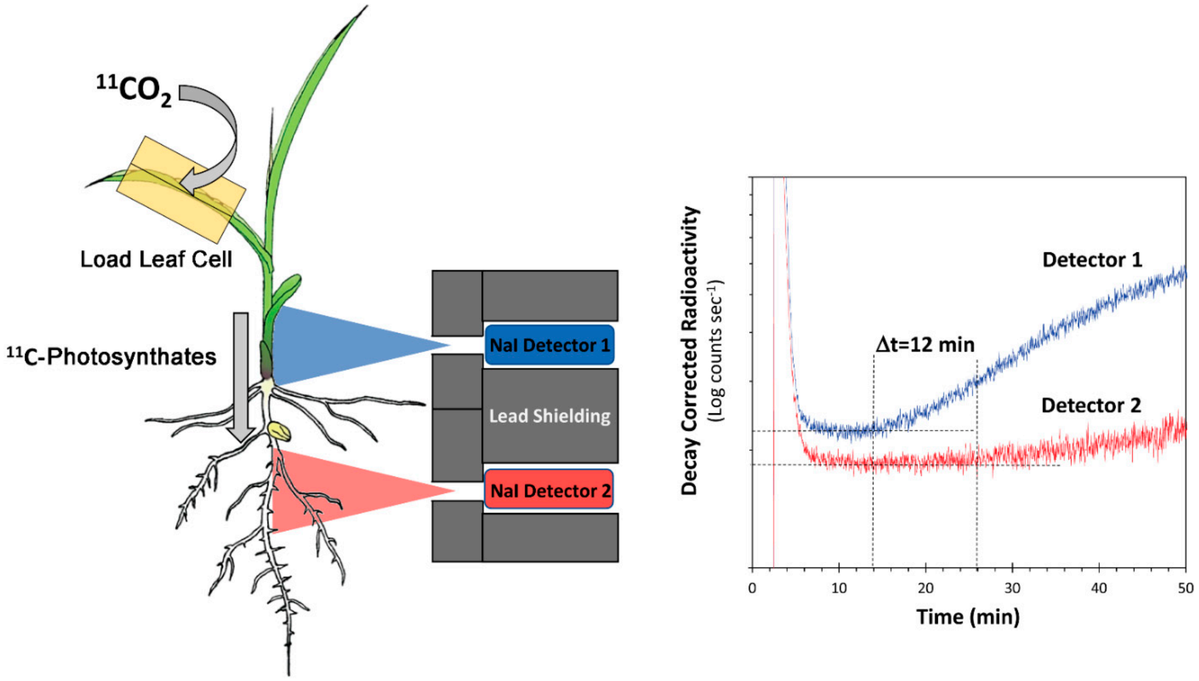

Figure S2. Experimental setup for measuring transport of ^{11}C-photosynthates across plant tissues.

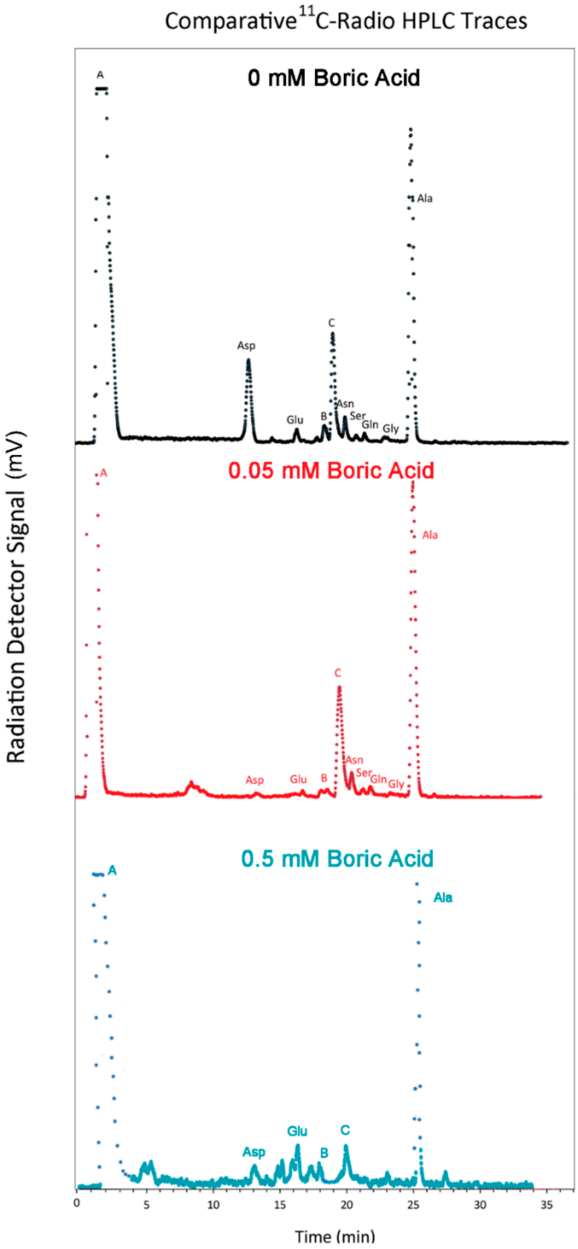

Figure S3. Radio HPLC trace showing elution of ^{11}C-amino acids and non-amino acid metabolites. Peaks designated by letters A, B & C were not amino acids but other unidentified radiolabeled metabolites that eluted the HPLC column. All data was decay corrected to a common timepoint and normalized to the same level of ^{11}C-alanine for comparison.

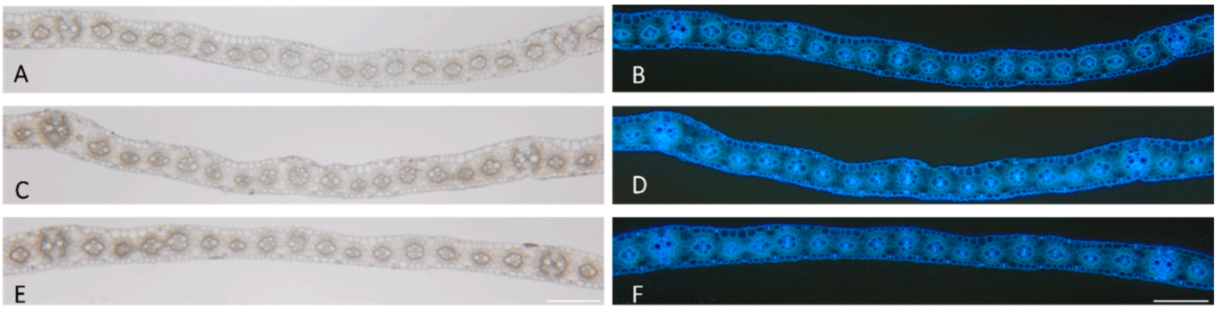

Figure S4. Cross sections of leaves showed no appreciable changes of leaf anatomy for plants grown under 0 mM boric acid (BA) and 0.5 mM BA treatments. Panels A and B; C and D; E and F are free hand leaf cross sections from plants grown under high boron concentration (0.5 mM BA), normal boron concentration (0.05 mM BA) and low boron concentration (0 mM BA), respectively. Panels A, B and C were imaged under Bright Field while Panels B, C, D were imaged under UV light. Scale bar = 200μm.
